# Karyotype and Gene Order Evolution from Reconstructed Extinct Ancestors Highlight Contrasts in Genome Plasticity of Modern Rosid Crops

**DOI:** 10.1093/gbe/evv014

**Published:** 2015-01-28

**Authors:** Florent Murat, Rongzhi Zhang, Sébastien Guizard, Haris Gavranović, Raphael Flores, Delphine Steinbach, Hadi Quesneville, Eric Tannier, Jérôme Salse

**Affiliations:** ^1^INRA/UBP UMR 1095 GDEC ‘Génétique, Diversité et Ecophysiologie des Céréales’, Clermont Ferrand, France; ^2^Faculty of Engineering and Natural Sciences, International University of Sarajevo, Sarajevo, Bosnia and Herzegovina; ^3^INRA ‘Unité de Recherche en Génomique et Informatique’, Centre INRA de Versailles, Versailles, France; ^4^INRIA Rhône-Alpes, Université de Lyon 1, CNRS UMR5558, Laboratoire Biométrie et Biologie Évolutive, Villeurbanne Cedex, France

**Keywords:** evolution, paleogenomics, dominance, polyploidy, plasticity

## Abstract

We used nine complete genome sequences, from grape, poplar, *Arabidopsis*, soybean, lotus, apple, strawberry, cacao, and papaya, to investigate the paleohistory of rosid crops. We characterized an ancestral rosid karyotype, structured into 7/21 protochomosomes, with a minimal set of 6,250 ordered protogenes and a minimum physical coding gene space of 50 megabases. We also proposed ancestral karyotypes for the Caricaceae, Brassicaceae, Malvaceae, Fabaceae, Rosaceae, Salicaceae, and Vitaceae families with 9, 8, 10, 6, 12, 9, 12, and 19 protochromosomes, respectively*.* On the basis of these ancestral karyotypes and present-day species comparisons, we proposed a two-step evolutionary scenario based on allohexaploidization involving the newly characterized A, B, and C diploid progenitors leading to dominant (stable) and sensitive (plastic) genomic compartments in any modern rosid crops. Finally, a new user-friendly online tool, “DicotSyntenyViewer” (available from http://urgi.versailles.inra.fr/synteny-dicot), has been made available for accurate translational genomics in rosids.

## Background

Fossil records and phylogenetic inference have indicated that flowering plants, or angiosperms, are derived from a common ancestor 150–250 Ma, during the early Cretaceous period (Friis et al. 2006; Moore et al. 2007). Modern flowering plants include socioeconomically important crop species from both the monocot (mostly grasses) and eudicot (mostly rosids) lineages. The monocot genome sequences available include sequences from three subfamilies of grasses (*Poaceae*)—the *Panicoideae* (sorghum, maize, millet), *Ehrhartoideae* (rice), and *Pooideae* (*Brachypodium*)—that diverged from a common ancestor 50–70 Ma (International Rice Genome Sequencing Project 2005; Paterson et al. 2009; Schnable et al. 2009; International Brachypodium Initiative 2010). Numerous paleogenomic studies using reconstructed ancestors have investigated genome paleohistory and established that grasses are derived from an ancestor with a haploid number (*n*) of 7 to 12 chromosomes. These ancestral grass karyotypes (AGKs) contained up to 16,464 ordered protogenes occupying a physical coding space of 33 Mb (Salse, Abrouk, Bolot, et al. 2009; Salse, Abrouk, Murat, et al. 2009; Murat et al. 2014). Present-day grass genomes have developed from the *n* = 12 ancestor through distinct, independent, and ancestral chromosome shuffling events (Bolot et al. 2009). The change in chromosome number in grasses, from the *n* = 12 of the common ancestor to the numbers present in modern species, has been shown to be driven by nonrandom centric break-mediated double-strand break repair events involving illegitimate centromeric/telomeric recombination between nonhomologous chromosomes, leading to nested chromosome fusions and synteny break points (Bolot et al. 2009; Murat et al. 2010). Ancestral grass polyploidization (transition from *n* = 7 to 12 in AGKs) was followed by a genome-wide diploidization (also referred to as partitioning) process involving the differential elimination of duplicated redundant genes. This gene loss after polyploidization did not occur randomly throughout the genome and led to the establishment of dominant (higher levels of duplicated gene loss) and sensitive (lower levels of duplicated gene loss) subgenomes in paleo- or neopolyploids (Murat et al. 2010, 2014; Schnable et al. 2012; Pont et al. 2013).

**Fig. 1.—:**
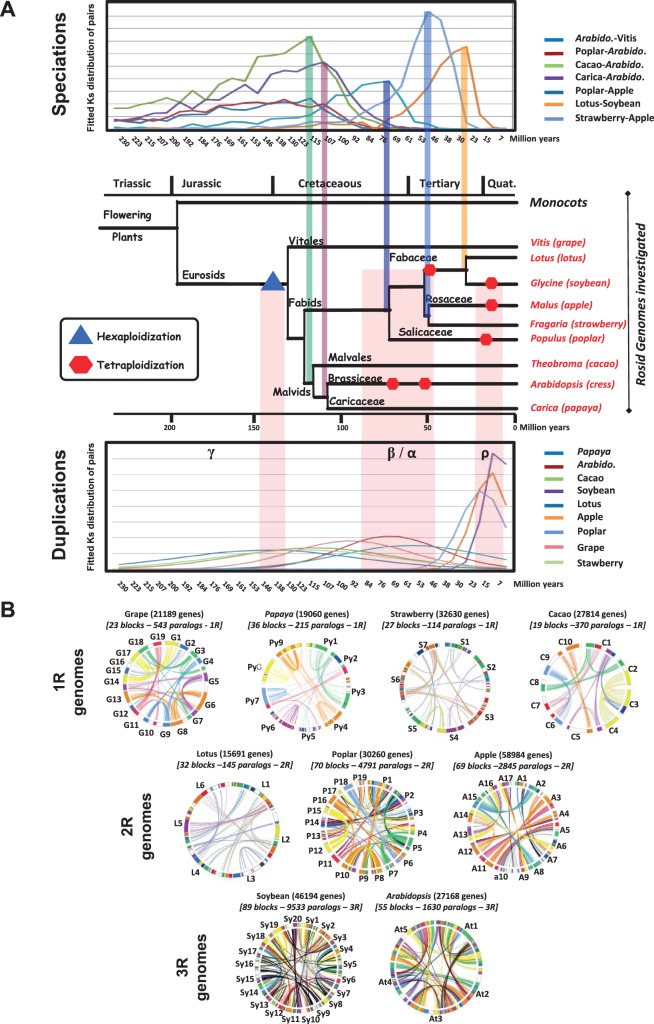
Rosid genome phylogeny, duplication, and synteny. (A) Rosid phylogeny. Schematic representation of the phylogenetic relationships between angiosperm species. Divergence times from a common ancestor are indicated on the branches of the phylogenetic tree (in million years), and the geological period (Jurassic, Cretaceous, Paleogene, and Neogene) is indicated at the top. WGD events are illustrated according to the color legend distinguishing hexaploidization and tetraploidization (left). Dating of speciation (top) and duplication (bottom) from fitted mixtures of log-normal distributions of duplicated Ks values are illustrated in the figure with a color code explained in the species legend at the right. (B) Rosid genome duplication and synteny. Schematic representation of the syntenic (blocks of the same color between genomes) and duplicated (blocks of the same color within genomes) regions identified in the grape (G1–19), papaya (Py1–9), strawberry (S1–7), cacao (C1–10), lotus (L1–6), poplar (P1–19), apple (A1–17), soybean (Sy1–20), and Arabidopsis (At1–5) chromosomes (in circles). Each line within the genome circles connects duplicated genes. The different colors of the blocks reflect their origins, from the seven ancestral protochromosomes.

Investigations of the paleohistory of modern eudicot genomes have also showed that these plants are derived from an *n* = 7 ancestor that underwent a paleohexaploidization event to generate an *n* = 21 intermediate (for a review, see Salse 2012). Unlike grasses, rosids underwent several species-specific duplication/triplication events that are still poorly understood, and contrasting models of their evolution have been proposed (for a review, see Van de Peer et al. 2009; Proost et al. 2011; Lee et al. 2013). Most previous studies have been based on classical phylogenetic investigations, often associated with the incorrect calibration of speciation/duplication events calculated in the presence of highly heterogeneous sequence substitution rates due to differences in evolutionary forces between gene families. However, the recent release of numerous eudicot genome sequences (for grape, Jaillon et al. 2007; poplar, Tuskan et al. 2006; *Arabidopsis*, AGI 2000; soybean, Schmutz et al. 2010; papaya, Ming et al. 2008; lotus, Sato et al. 2008; apple, Velasco et al. 2010; strawberry, Shulaev et al. 2011; cacao, Argout et al. 2011; table 1 and fig. 1*A*) has opened up new possibilities for studies of the paleohistory of these species, in terms of ancestral shared and recent species-specific duplication events and ultimately ancestral karyotype structures (i.e., chromosome and gene numbers/orders).

**Table 1:** Rosid Genome Data Sets Used for this Paleogenomics Study

Ancestor	Species	Common Name	Chromosome	Genome size (Mb)	Annotated genes	Synteny	Duplication	Chromosome equation	WGD
Dicot pre-WGD ancestor	–	–	7	–	626 ordered protogenes	–	–	7	**0R**
Dicot post-WGD ancestor	–	–	21	–	6,250 ordered protogenes	–	–	7 × 3	**1R**
	*Vitis vinifera*	Grape	19	302	21,189	Reference	543 - 23 - 71	21+2−4	1R
	*Carica papaya*	Papaya	9	234	19,060	3199 - 65 - 75	215 - 36 - 55	21+6−18	1R
	*Theobroma cacao*	Cacao	10	218	27,814	4472 - 21 - 81	370 - 19 - 66	21+2−13	1R
	*Arabidopsis thaliana*	Thalecress	5	119	33,198	2389 - 80 - 99	1630 - 55 - 83	8+4−7	3R
Fabaceae ancestor	–	–	6	–	861 ordered protogenes	–	–	21+1−16	1R
Papilionoideae ancestor	–	–	12	–	1,159 ordered protogenes	–	–	6 **×** 2	1R
	*Glycine max*	Soybean	20	949	46,194	4013 - 164 - 97	9533 - 89 - 55	(6 × 2 × 2) +13-17	3R
	*Lotus japonicus*	Lotus	*6*	462	15,691	1720 - 80 - 61	145 - 32 - 35	(6 × 2)+0−6	2R
Rosaceae ancestor	–	–	*9*	–	2,672 ordered protogenes	–	–	21+3−15	1R
	*Fragaria vesca*	Strawberry	7	208	32,630	3289 - 94 - 70	114 - 27 - 19	9+0−2	1R
	*Malus domestica*	Apple	17	528	58,984	3498 - 104 - 70	2845 - 69 - 59	(9 × 2)+4−5	2R
Salicaceae ancestor	*–*	*–*	12	–	3,196 ordered protogenes	–	–	21+6−15	1R
	*Populus trichocarpa*	Poplar	19	307	30,260	4555 - 87 - 92	4164 - 46 - 73	(12 × 2)+4−9	2R
					Eudicots total	27135-695-81	19559-396-57		

A bias in the loss of duplicated genes following polyploidization has been reported in a few species, as part of the genome rearrangements occurring during the course of the paleohistory of eudicots. Ziolkowski et al. (2003) and Henry et al. (2006) reported a higher rate of gene deletion in one of the duplicated segments resulting from two rounds (R) of whole-genome duplication (WGD) in *Arabidopsis* dating back to 24–40 and 65 Ma. Cheng et al. (2012) and Roulin et al. (2013) recently reported a similar pattern in biased gene retention/deletion following the hexaploidization of *Brassica rapa* (13–17 Ma) and the tetraploidization of soybean (13 and 59 Ma), respectively. Genome partitioning has been investigated and shown to have occurred in a few eudicot species, in relation to lineage- and even species-specific WGD, but this subgenome dominance phenomenon has not yet been investigated in relation to the shared ancestral hexaploidization event (known as γ) potentially affecting all modern rosid crops, which occurred ∼150 Ma.

In this study, we used nine genomes 1) to reconstruct the paleohistory of rosids from their founder ancestral rosid karyotypes (ARKs), precisely characterized in terms of their protochromosome and protogene contents, 2) to determine the nature, origin, and timing of shared and lineage-specific polyploidization events, 3) to decipher the general pattern of ancestral subgenome dominance as part of a general polyploidization-driven diploidization process, leading to the definition of a new two-step evolutionary model, and finally 4) to develop an applied tool (i.e., online “DicotSyntenyViewer” platform) for accurate translational genomics from models to rosid crops.

## Materials and Methods

### Genome Sequences

The sequences of the nine rosid genomes were downloaded from the public PHYTOZOME (http://www.phytozome.net/, last accessed February 13, 2015) website of the Joint Genome Institute (California, USA). The genomes studied were those of grape (19 chromosomes, 302 Mb, 21,189 genes; Jaillon et al. 2007), poplar (19 chromosomes, 294 Mb, 30,260 genes; Tuskan et al. 2006), *Arabidopsis* (5 chromosomes, 119 Mb, 33,198 genes; AGI 2000), soybean (20 chromosomes, 949 Mb, 46,195 genes; Schmutz et al. 2010), papaya (9 chromosomes, 234 Mb, 19,060 genes; Ming et al. 2008), lotus (6 chromosomes, 462 Mb, 15,691 genes; Sato et al. 2008), apple (17 chromosomes, 528 Mb, 58,984 genes; Velasco et al. 2010), strawberry (7 chromosomes, 208 Mb, 32,630 genes; Shulaev et al. 2011), and cacao (10 chromosomes, 218 Mb, 27,814 genes; Argout et al. 2011). For reconstruction of the intermediate ancestor of the Rosaceae, we also included the genomes of *Prunus mume* (8 chromosomes, 230 Mb, 27,852 genes; Zhang et al. 2012), pear (7 chromosomes, 512 Mb, 42,812 genes; Wu et al. 2013), and an Expressed Sequence Tag (EST)-based genetic map and draft genome of peach (784 markers, 8 chromosomes, 265 Mb, 27,852 genes; IPGI 2013).

### Ancestral Chromosome Reconstruction

Orthologous and paralogous genes (based on a cumulative identity percentage [CIP] of 60% and a cumulative alignment length percentage [CALP] of 70%) and blocks (based on Closeup software, with a density ratio [DR] of 2, a cluster length [CL] of 20, and a match number of 5) were identified as described by Salse, Abrouk, Bolot, et al. (2009) and Salse, Abrouk, Murat, et al. (2009), figure 2. Ancestral karyotypes were reconstructed as described by Murat et al. (2012, 2014), by comparing the blocks duplicated or conserved between two genomes (derived from the validated orthologous genes/blocks) and within a single genome (derived from the validated paralogous genes/blocks) to define contiguous ancestral regions (CARs). Briefly, paralogous blocks within two different genomes but located in orthologous positions within these two genomes were considered 1) unique in the ancestor (i.e., a CAR) and 2) derived from a shared prespeciation duplication event. In contrast, paralogous blocks present in one genome and not associated with duplicated regions in orthologous positions within the other genomes investigated were considered 1) to correspond to a species-specific duplication and 2) to be derived from a postspeciation duplication event (Murat et al. 2012). On the basis of the CARs identified, we determined the most likely evolutionary scenario based on the following assumptions: 1) Ancestor modeling was based on duplications (or shuffling events) at orthologous positions in modern species, which were therefore considered to be ancestral and 2) evolutionary history was considered to correspond to the smallest number of shuffling operations (including inversions, deletions, fusions, fissions, translocations) that could account for the transition from the reconstructed ancestral genome to modern karyotypes (Murat et al. 2012, 2014).

### Ancestral Gene Order Reconstruction

Ancestral gene order within CARs was inferred by a generalization of the method implemented in ANGES software (Jones et al. 2012), adapted for possible massive gene losses (Gavranovic et al. 2011). We implemented the tools described above according to three different principles, according to the nature of the ancestor sought: An ancestor preceding a speciation, preceding a WGD, or preceding the ancestral hexaploidization (see below).

(1) We used gene orthology relationships between 1R genomes to reconstruct the order of genes in ancestors preceding a speciation (e.g., the malvid ancestor, and the common ancestor of malvids and fabids). An ancestral marker was defined as an informative family of genes found to be orthologous between species and an adjacency of ancestral markers was defined as a pair of ancestral markers found to be contiguous in at least two informative species. A common interval of ancestral markers is a set of ancestral markers found to be contiguous (but present in any order) in at least two informative species. As in ANGES (Jones et al. 2012), we reconstructed all adjacencies and maximal common intervals between informative pairs of genomes. We then used a method similar to that of Gavranovic et al. (2011) to construct a matrix in which the columns corresponded to the ancestral genes, with each row corresponding to a common interval. We entered “1” in the matrix if the gene was part of the interval considered, “0” if the gene was present in the two genomes compared but not part of the interval considered, and “X” in all other cases. We ordered the columns (thereby ordering the ancestral genes) such that, in each row, there was never a 0 between two 1 values (the matrix sandwich problem; Gavranovic et al. 2011). No parameters were used because the initial markers were the genes themselves, and no synteny blocks were constructed, and the definition of adjacencies and common intervals were strict, allowing no flexibility.

(2) We reconstructed the genomes of ancestors preceding a WGD (i.e., for Malpighiales, Rosaceae, Papilionoideae, and soybean), by applying the “double conserved synteny” (DCS) principle used, for example, by Kellis et al. (2004), and by Ouangraoua et al. (2011) for the analysis of synteny relationships in yeasts in the context of WGDs. We used the software of Ouangraoua et al. (2011), with all flexibility parameters set to 0. The input for this software is a list of genes orthologous between a 1R genome and a 2R genome, or between a 2R genome and a 3R genome. The output is all segments of contiguous genes of the 1R (or 2R) genome for which two orthologous gene segments are present in the 2R (or 3R) genome. The results are then filtered according to a statistical test of significance of these segments (Ouangraoua et al. 2011). The segments identified were then used as ancestral markers. Each ancestral marker was present once in the 1R (or 2R) genome, and twice in the 2R (or 3R) genome. We computed the adjacencies and common intervals of these segments as defined above. The segments were then ordered with ANGES (Jones et al. 2012), using a 0/1 matrix, as before (but this time with no X values in the matrix because the markers were present in the genomes considered), and the conserved segments were ordered. See Gavranovic et al. (2011) and Ouangraoua et al. (2011) concerning the validation and robustness of these methods.

(3) There is currently no method for reconstructing gene order for a chromosome that has undergone triplication (such as the paleohexaploidization occurring early in the evolution of rosids/eudicots). Nakatani et al. (2007), Jaillon et al. (2004), and Kohn et al. (2006) reconstructed ancestral karyotypes after two rounds of WGD in early vertebrate evolution, but none of the methods they used determined the order of the genes. Our reconstruction method was based on a comparison of gene order along the three paralogous chromosomes in the 1R genomes arising from the triplication. This approach made it possible to compare chromosomes two-by-two. It was previously used by Murat et al. (2012, 2014) to retrieve the ancestral order of grass genes after the ancestral WGD, and it provides an initial insight into gene order. However, more information can be obtained by making use of the specificity of hexaploidization, and examining all three chromosomes together. According to the principle of DCS, there should be one segment present in the nonduplicated genome, and two orthologs in the duplicated genome. We applied this principle, as implemented by Ouangraoua et al. (2011), to identify segments present once on one chromosome but with two paralogs on the other two chromosomes. We did this by extracting all the paralogous pairs of genes in the 1R genomes, each of these pairs defining an ancestral gene. The input for the DCS method is usually pairs of orthologous genes (see above). We provided the paralogous pairs identified as the input, and set all flexibility parameters to 0. This generated a set of triplets of paralogous segments, defining the ancestral gene intervals. These intervals were then assembled, by defining the following matrix, taking into account the constraints of the matrix sandwich problem: For each segment identified, we entered a value of 1 if an ancestral gene was present in the segment, 0 if the gene was present on the chromosome compared, but not in the segment, and X otherwise. Statistical tests were performed (with DCS software) to assess the significance of the paralogous segments.

Blocks of ordered ancestral genes were then mapped onto the previously reconstructed ancestral chromosomes defining linked (mapped and oriented on the ancestral chromosomes) and unlinked (small blocks of reordered ancestral genes unmapped on the ancestral chromosomes) ancestral ordered genes.

### Subgenome Partitioning Analysis

For each triplet of ancestral chromosomes, we determined the number of genes retained (i.e., conserved between species and/or ancestrally duplicated) on dominant and sensitive chromosomes, to model the partitioning and variance of retained triplicated genes without subgenome dominance (H_0_: Triplicated gene deletion is random between paralogous chromosomes). We then carried out chi-square tests to compare the observed value (the number of genes retained in triplicated blocks) and the expected value (assuming an equal distribution of retained duplicated genes between two blocks). For each triplet, A1, A2, and A3, we compared each pair—A1 and A2, A2 and A3, and A1 and A3—in a binomial test *B*(*n*,*p*) in which *n* = *n*1 + *n*2, *n* = *n*2 + *n*3, *n* = *n*1 + *n*3, and *p* = ½. If the *p* value obtained was lower than 0.005, we rejected the null hypothesis and considered the expected and observed values to be significantly different. In this case, the biased retention of triplicates or subgenome dominance was considered to be statistically validated, with a significant difference in the pattern of ancestral gene retention between the two ancestral chromosomes.

### Dating of Speciation and Duplication Events

We performed classical sequence divergence analysis, together with speciation and duplication event dating analysis based on a comparison of the rates of nonsynonymous (*K_a_*) and synonymous (*K_s_*) substitutions. A mean substitution rate (*r*) of 6.5 × 10^−^^9^ substitutions per synonymous site per year is classically applied to calibrate the ages of the paralogous and orthologous genes considered (Gaut et al. 1996; SanMiguel et al. 1998). The time (*T*) is then estimated using the formula *T* = *K_s_*/2*r*. The *K_s_* between paralogs has been modeled as a mixture of log-transformed exponential and normal distributions, representing recent and ancient WGDs. The distribution of *K_s_* can thus be described as a mixture of log-normal components representing single or multiple rounds of genome duplication, with EMMIX software (http://www.maths.uq.edu.au/∼gjm/emmix/emmix.html, last accessed February 13, 2015). We followed this procedure and then selected the best mixed model for each round of duplication on the basis of the Bayesian information criterion and an additional constraint relating to the mean/variance structure for *K_s_* (Cui et al. 2006).

## Results

### Conserved and Duplicated Genes in Rosids

**Fig. 2.—:**
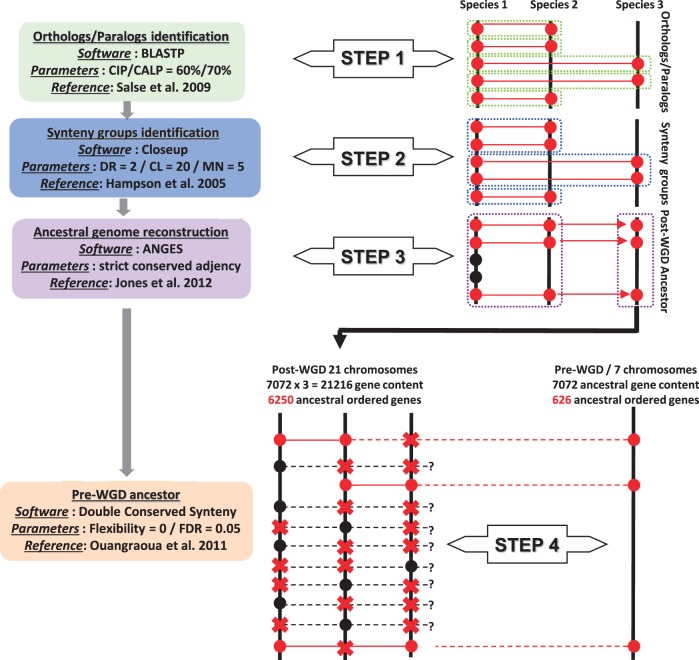
Method for ancestral rosid genome reconstruction. A four-step method was used. STEP 1: Ortholog/paralog identification based on the application of CIP (Cumulative Identiy Percentage)/CALP (Cumulative Alignment Length Percentage) parameters in BLASTP, with thresholds of 60% and 70%, respectively. STEP 2: Synteny block identification with Closeup, with DR (Density Ratio) = 2, CL (Cluster Length) = 20, MN (Match Number) = 5. STEP 3: Ancestral genome reconstruction with ANGES, on the basis of strict gene adjacency conservation. STEP 4: Illustration of the reconstruction of the prehexaploidization (7 chromosomes) ancestor from the reconstructed posthexaploidization ancestor (21 chromosomes) based on 7,072 conserved genes (potentially the ancestral gene content) and the 626 remaining retained duplicates/triplicates. Pre-WGD ancestors were reconstructed on the basis of DCS (Double Conserved Synteny), with flexibility = 0 and a false discovery rate of 0.05.

The synteny of rosids—as exemplified by grape, poplar, *Arabidopsis*, soybean, lotus, apple, strawberry, cacao, and papaya, representing the Vitales, the fabid and malvid subfamilies, in which genome size may vary by a factor of up to 10 (fig. 1*A* and table 1)—was reassessed by defining conserved/duplicated gene pairs (on the basis of alignment parameters and statistical tests) and block pairs (using Closeup software), as described by Salse, Abrouk, Murat, et al. (2009) and illustrated in figure 2 (green and blue panels, respectively). Orthologs and paralogs were selected on the basis of a *K_s_* filtering procedure, such that the pairs selected corresponded to known speciation and polyploidization events (ρ, α, β, γ; fig. 1*A*, speciation and duplication panels). We provide an updated and more exhaustive multispecies repertoire of orthologs (27,135 pairs defining 695 syntenic blocks covering 81% of the genome on average) and paralogs (19,559 pairs defining 396 blocks covering 57% of the genome on average) for rosids (fig. 1*B* and table 1) than previous studies (Salse 2012; Murat et al. 2012). Finally, the conserved chromosome-to-chromosome syntenic relationships characterized between grape “G,” poplar “P,” *Arabidopsis* “At,” soybean “Sy,” lotus “L,” apple “A,” strawberry “S,” cacao “C,” and papaya “Py” are shown as a color code on the nine genome circles in figure 1*B* and in supplementary table S1, Supplementary Material online.

**Fig. 3.—:**
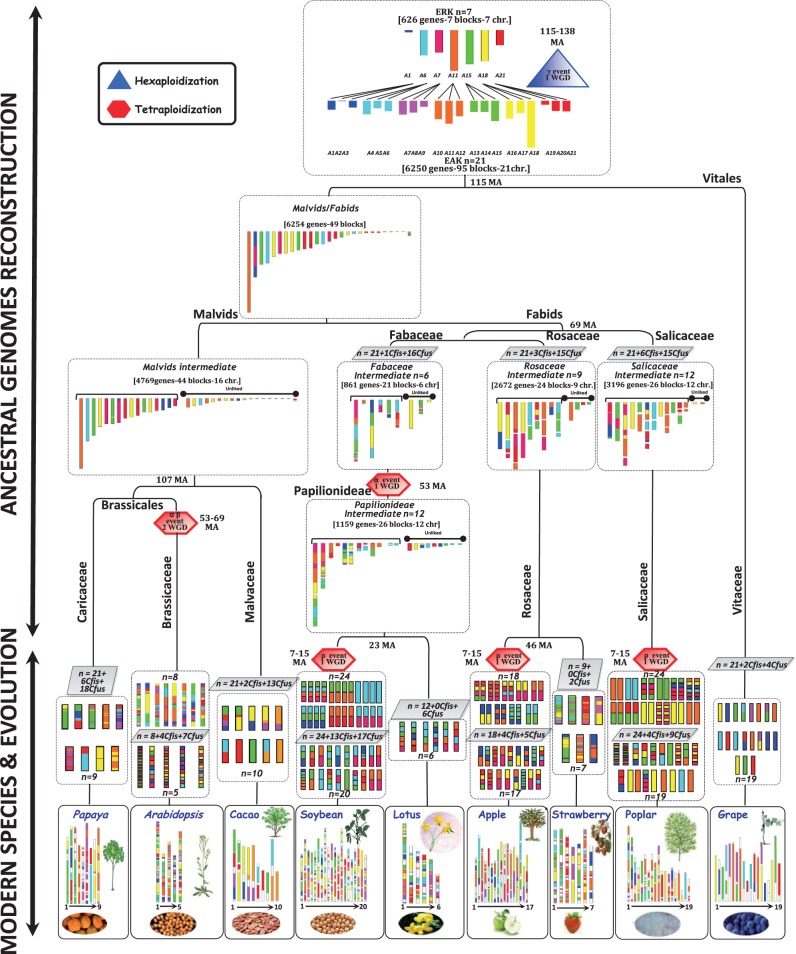
Scenario for the evolution of rosid genomes from reconstructed extinct ancestors. The rosid chromosomes are represented with color codes to illustrate the evolution of segments from a common ancestor with seven protochromosomes (named according to the grape nomenclature, i.e., A1, A4, A7, A10, A13, A16, A19). The “R” events that have shaped the structure of the different rosid genomes during their evolution from the ARK are indicated as ρ (species-specific WGD), α–β (ancestor intermediate or lineage-specific WGD), and γ (ancestral shared WGD). The present-day structure of the nine rosid genomes is represented at the bottom of the figure. The various shuffling events, such as chromosomal fusions (*Cfus*) and fissions (*Cfis*) are indicated within boxes. The ancestral reconstructed karyoptypes (ARK *n* = 7 and *n* = 21) and the lineage-specific intermediates reconstructed for the prespeciation malvids/fabids, malvids, Salicaceae, Rosaceae, Fabaceae, and Papilionideae are illustrated with a seven-color code (reflecting the structure of the ARK). Unlinked blocks correspond to reconstructed ancestral blocks that could not be associated with the characterized ARK protochromosomes.

Integration of the previously described blocks duplicated within species and syntenies between species for the nine rosid genomes investigated made it possible to characterize precisely the seven known shared ancestral triplicated blocks (Jaillon et al. 2007; Salse 2012). These seven ancestral triplicated blocks, derived from the shared paleohexaploidization event (referred to as γ), are spread throughout the genomes of grape, poplar, *Arabidopsis*, soybean, lotus, apple, strawberry, cacao, and papaya and correspond to the following known chromosomal relationships for the grape (G) reference genome: G1-G14-G17/G2-G15-G12-G16/G3-G4-G7-G18/G4-G9-G11/G5-G7-G14/G6-G8-G13/G10-G12-G19 (fig. 1*B*, color code). The identification of 1) at least remnants of the hexaploidy event (i.e., inferred duplication) and 2) seven conserved ancestral chromosome blocks (i.e., synteny inference) confirmed an *n* = 21 (3 × 7) ancestral intermediate common to all rosid genomes investigated (fig. 3). The rosid families then underwent different rounds (fig. 1*A*–*B*) of species-specific paleopolyploidization events (ρ, α, and β) and ancestral chromosome fusions/fissions (*Cfus* for chromosome fusions and *Cfis* for chromosome fissions) to achieve their modern genome structures, as established below.

### Reconstruction of ARKs

We used ANGES software (Jones et al. 2012), together with the strategy described by Gavranovic et al. (2011), to propose a gene order for the ancestral genomes of the Caricaceae, Brassicaceae, Malvaceae, Fabaceae, Rosaceae, Salicaceae, and Vitaceae, including the pretriplication rosid ancestor (*n* = 7 pre-γ). No published method has ever been reported to reconstruct the gene order of an ancestral genome in the context of hexaploidization. We used DCS to unmask synteny signals in the context of WGDs (through integration of the previously identified paralogous and orthologous blocks), and the “matrix sandwich” method (Gavranovic et al. 2011; Jones et al. 2012), to order genes within protochromosomes (see Ancestral Gene Order Inference; fig. 2, purple and brown panels). With this strategy, we were able to reorder 6,250 protogenes (from a total of 7,072 genes conserved in all nine genomes investigated; fig. 3, top) covering 21 protochromosomal groups corresponding to the paleohexaploid (post-γ) ancestor (ARK) (see supplementary tables S2–S4, Supplementary Material online). Only 626 ancestral genes retained as duplicates/triplicates in the posthexaploidization ancestor could be accurately reordered in the 7 prehexaploidization protochromosomes from a putative ancestral gene pool of a minimum of 7,072 protogenes. From the retained ARK structure, the grape genome underwent 2 *Cfis* and 4 *Cfus* events to reach the 19 chromosomes of modern varieties (fig. 3).

We used the same strategy to reconstruct the genome of the malvid/fabid prespeciation ancestor (an ancestor common to all the genomes investigated other than grape), consisting of 6,254 ordered genes organized into 49 ancestral blocks. The malvid ancestor (based on a comparison of papaya, *Arabidopsis,* and cacao) had 4,769 protogenes delineating 44 blocks, which merged (through mapping on protochromosomes) into 16 protochromosomes. The modern papaya (9 chromosomes = 21(ARK) + 6*Cfis−*18*Cfus*) and cacao (10 chromosomes = 21(ARK) + 2*Cfis−*13*Cfus*) plants were derived from the malvid ancestor without additional polyploidization, whereas the *Arabidopsis* genome underwent duplication (α, β) during the evolution of the Brassicaceae ancestor, which had eight chromosomes, followed by four *Cfis* and seven *Cfus* events, to attain its modern *n* = 5 genome structure. The Salicaceae (corresponding to the preduplication poplar genome) had 3,196 protogenes organized into 26 blocks assembled into 12 protochromosomes (21(ARK) + 6*Cfis−*15*Cfus*). The modern poplar genome was derived by duplication (ρ) of the *n* = 12 Salicaceae intermediate, followed by four *Cfis* and nine *Cfus* events. The Rosaceae ancestor (based on a comparison of apple and strawberry) had 2,672 genes located in 24 blocks, defining 9 protochromosomes (21(ARK) + 3*Cfis−*15*Cfus*). The modern strawberry genome was derived from the *n* = 9 Rosaceae ancestor (with two *Cfus* events), whereas the apple underwent a tetraploidization (*n* = 18 intermediate) event (ρ), followed by four *Cfis* and five *Cfus* events. The Papilionideae (based on a comparison of soybean and lotus) ancestor was reconstructed with 1,159 protogenes (in 26 blocks defining 12 protochromosomes) that underwent 6 *Cfus* events to yield the modern lotus genome. In contrast, the modern soybean genome was derived from a duplication of the genome of the *n* = 12 Papilionideae (i.e., postpapilionoid WGD state) ancestor (ρ, *n* = 24 intermediate), followed by 13 *Cfis* and 17 *Cfus* events. Finally, the soybean and lotus genomes experienced a shared tetraploidization event (α). This made it possible to reconstruct the genome of a Fabaceae ancestor (corresponding to the preduplication Papilionideae genome), consisting of 861 protogenes mapping to 21 blocks defining 6 protochromosomes (i.e., prepapilionoid WGD state; fig. 3). The current ancestral Fabaceae karyotype, derived from the reconstruction of an *n* = 6 prepapilionoid WGD and an *n* = 12 postpapilionoid WGD intermediate, may be refined in the future, once genome sequences for the Cercideae, Detarieae, Dialiineae, and Duparquetia clades become available (Doyle 2012; Cannon et al. 2015). These integrative, multispecies investigations of the evolution of rosid crops made it possible to date of the major duplication and speciation events more precisely, as reported in figure 3 (dating, in millions of years, on the tree branches) and in additional supplementary table S5, Supplementary Material online, for the ρ (7–15 Ma), α and β (53–69 Ma), and γ (115–138 Ma) events.

### A Two-Step Theory of Rosid Genome Partitioning following Polyploidization

**Fig. 4.—:**
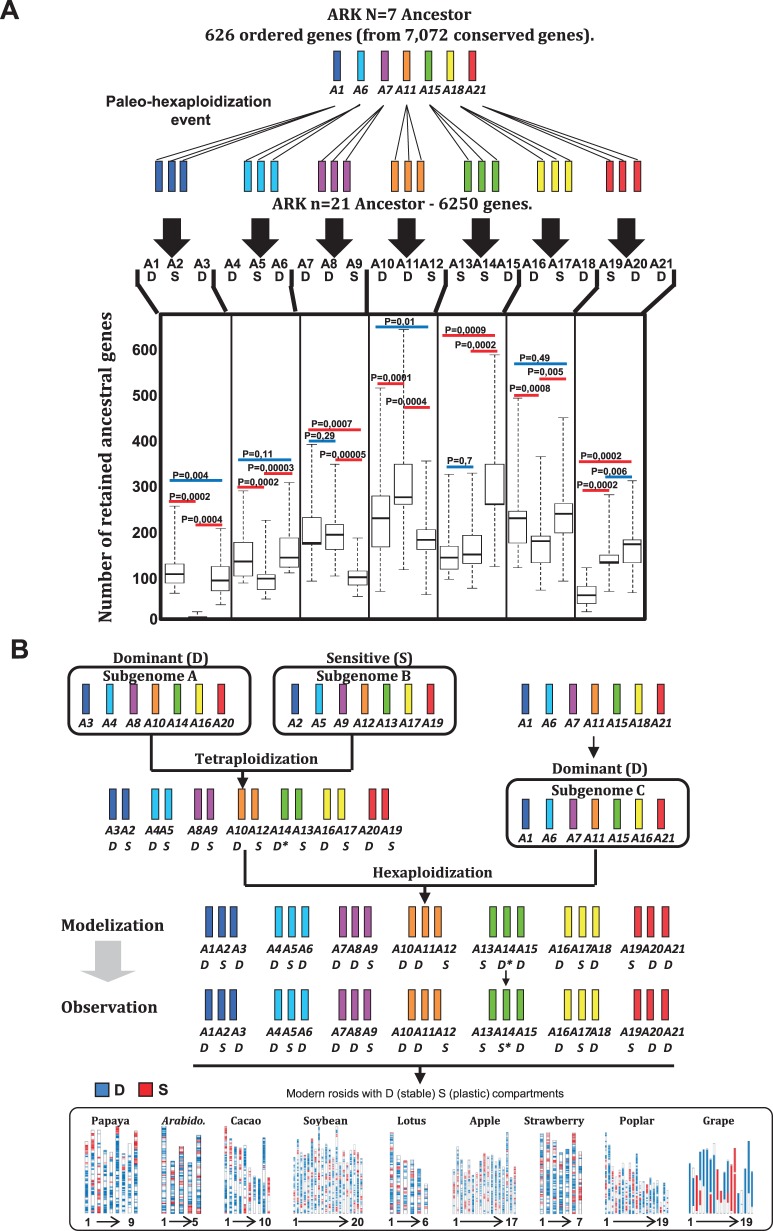
Rosid genome partitioning following paleohexaploidization. (*A*) Rosid subgenome dominance. The number of orthologous genes identified for each triplicated block (illustrated with the same color code), for the nine rosid genomes (left) investigated, is illustrated with dot boxes (*x* axis for the 21 ancestral blocks and *y* axis for the number of retained orthologous genes). Chromosome triplets displaying no significant differences (*P* > 0.005) in orthologous/ancestral gene retention are underlined in blue, whereas those displaying significant differences are underlined in red (*P* ≤ 0.005). “R” refers to rounds of WGD. (*B*) Two-step evolutionary theory. Illustration of the proposed evolutionary scenario responsible for shaping the 21 ancestral chromosomes following the hexaploidization of the seven-chromosome ARK, according to the subgenome dominance mechanism based on subgenomes A (A3-4-8-10-14-16-20), B (A2-5-9-12-13-17-19), and C (A1-6-7-11-15-16-21). This evolutionary model explains the observed differences in ancestral gene retention (between dominant “D” and sensitive “S” ancestral chromosomes) for 20 of the 21 ancestral blocks, the exception being A14 (indicated by a black star).

We used the reconstructed ARKs (a posthexaploidization ancestor with 6,250 protogenes and 21 protochromosomes and a prehexaploidization ancestor with 626 protogenes and 7 protochromosomes) to investigate the fate of the ancestral triplicates (arising from the paleohexaploidization). We determined whether the genes concerned were deleted or retained during the course of rosid evolution (fig. 4*A,* top). Figure 4*A* (bottom) illustrates the conservation of ancestral genes in the seven triplicated blocks from the modern genomes (expressed as the mean number of ancestral genes retained per block for the nine genomes investigated). We observed a bias in gene content (*P* ≤ 0.005 in binomial tests comparing the observed and simulated retention of ancestral genes in the triplicated blocks, see Materials and Methods). We were therefore able to distinguish precisely between dominant (D, higher levels of ancestral gene retention) and sensitive (S, higher levels of ancestral gene loss) ancestral and modern chromosomes. In this context, A1-3-4-6-7-8-10-11-15-16-18-20-21 appear to be dominant (D) blocks, whereas A2-5-9-12-13-14-17-19 appear to be sensitive (S) blocks.

We propose a new evolutionary scenario (a two-step theory) for the formation of the 21 ancestral chromosomes following the hexaploidization of the 7 ARK chromosomes, based on a subgenome dominance mechanism (fig. 4*B*). We suggest that hexaploidy resulted from an initial tetraploidization event (first step) between subgenomes A (A3-4-8-10-14-16-20) and B (A2-5-9-12-13-17-19), with A as the dominant subgenome with a higher level of ancestral gene retention and B as the sensitive subgenome prone to massive protogene deletion after hybridization. The initial tetraploidization event was followed by the hybridization (second step) of a third subgenome, subgenome C (A1-6-7-11-15-16-21), which appears to be dominant because it would have had a shorter evolutionary time available for gene loss or rearrangement in general (Malacarne et al. 2012). This homoeologous block fractionation predates rosid speciation (i.e., it is, by definition, ancestral) as the dominant and sensitive compartments have been maintained as orthologs between modern rosid genomes. This evolutionary model accounts for the observed differences in retention of the ancestral gene, for 20 of the 21 ancestral chromosomes, the only exception being A14, which appears to be sensitive in modern genomes (fig. 4*A*) but dominant in our evolutionary scenario (fig. 4*B*, black star). The origin of ancestral rosids, according to the two-step theory involving A, B, and C progenitors with postpolyploidization subgenome dominance, makes it possible to identify dominant (stable) and sensitive (plastic) compartments in any modern rosid crop, as illustrated in figure 4*B* (bottom).

### Rosid Crop Circles and a Synteny Viewer Tool

**Fig. 5.—:**
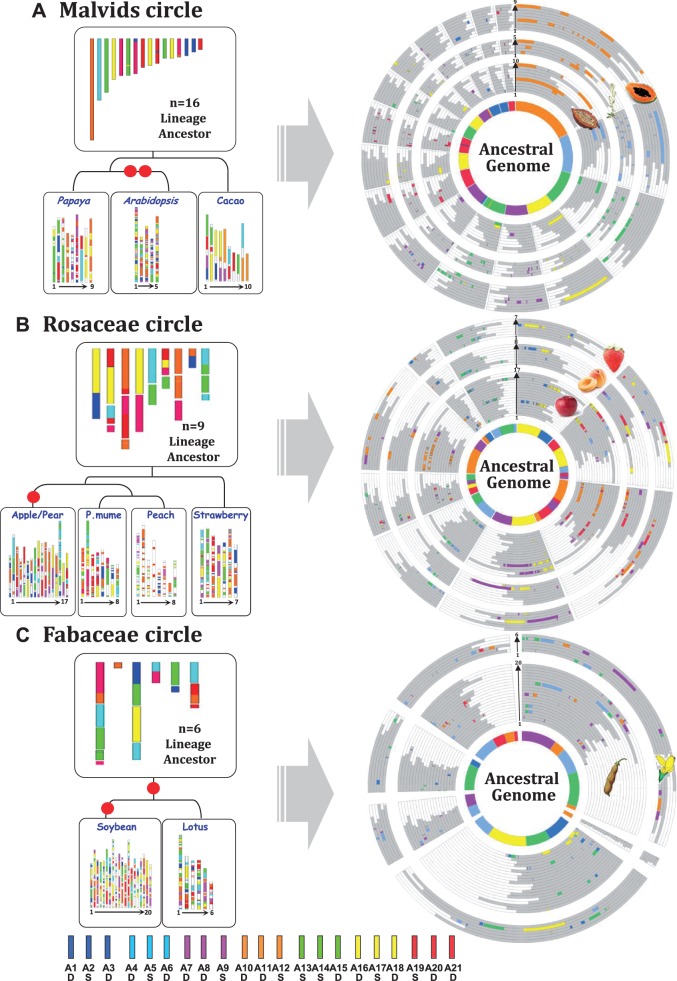
Rosid crop circles. Illustration of the lineage-specific ancestral karyotypes and the modern genomes (as a colored mosaic reflecting the chromosomal origin from founder protochromosomes) on the left, with chromosome-to-chromosome synteny relationships shown as concentric circles, on the right, for the malvids (*A*), Rosaceae (*B*), and Fabaceae (*C*).

The syntenic relationships between plant genomes have classically been illustrated through the use of circular consensus genetic maps, known as “crop circles,” as developed by Mike Gale and coworkers (Moore et al. 1995; Devos 2005) for grasses. In this approach, the genomes are arranged as concentric circles, with the size of each circle depending on the size of the corresponding genome. Taking into account the reconstructed ARK and the synteny and duplication relationships observed in modern rosid genomes, we generated crop circles for malvids (based on papaya, *Arabidopsis,* and cacao comparisons), Rosaceae (based on previous apple and strawberry comparisons and including published structurally related genomes of *Prunus* [Zhang et al. 2012], pear [Wu et al. 2013], and peach [IPGI 2013]) and Fabaceae (based on soybean and lotus comparisons). On the basis of this representation of chromosome-to-chromosome conserved synteny relationships (illustrated with a color code and with the ancestral karyotype structures as the innermost circles), it is possible to identify, for crop circles of any radius, the ancestral relationships and origins (WGD, breakages, fusions) of the different chromosomes in each of the modern malvid, Rosaceae, and Fabaceae genomes (fig. 5).

**Fig. 6.—:**
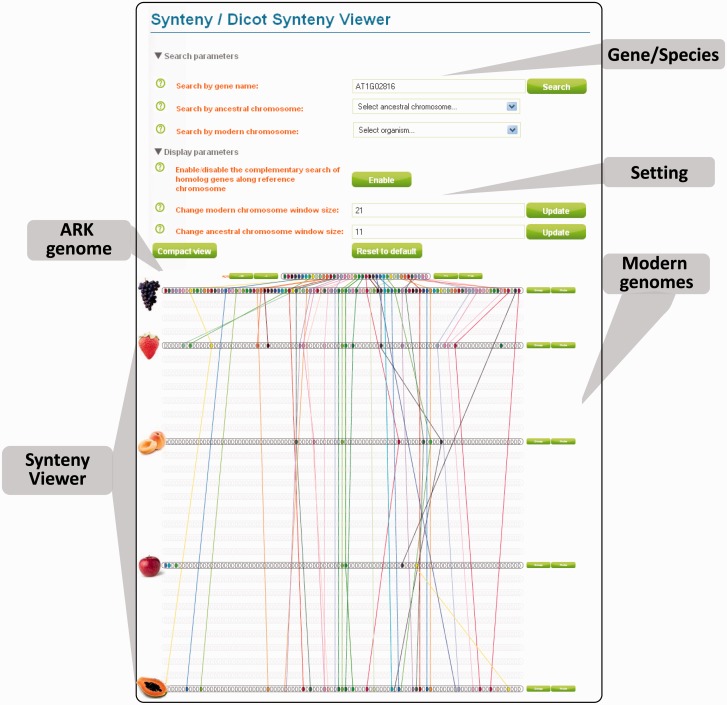
Rosid synteny viewer. The entry page of the DicotSyntenyViewer tool showing the settings (search by gene name, ancestral or modern chromosomes), including paleogenomic data visualization with the ARK (A1 is used as the example on this screen), modern rosid chromosomes from grape, poplar, *Arabidopsis*, soybean, lotus, apple, strawberry, cacao, papaya, and gene conservation (colored connecting lines) at the bottom. The Rosid synteny viewer tool is available from http://urgi.versailles.inra.fr/synteny-dicot (last accessed February 13, 2015) and can be used to navigate between rosid genomes through gene/species queries and use of the different setting parameters.

Finally, the paleogenomic data presented here, in terms of protochromosome characterization and the inference of protogene order in the ancestral genome structure, can now be considered an applied tool for accurate navigation between rosid genomes and for the transfer of genomic information (i.e., gene structures and functions) from models (such as *Arabidopsis*) to crop species of agronomic interest (such as trees, legumes, and crucifers). We have developed a user-friendly web tool called “PlantSyntenyViewer” (http://urgi.versailles.inra.fr/synteny-dicot, last accessed February 13, 2015), providing information about the orthologous, paralogous, and ancestral relationships described in this article. With this tool, it is possible to navigate between genomes, using a gene name, a modern chromosome nomenclature, or ancestral protochromosome references. This tool provides, for the first time, in a single screen, the complete set of orthologs and paralogs from the sequenced rosid genomes identified for any region or gene of interest considered (fig. 6).

## Discussion

Following the publication of the grape genome in 2007 (Jaillon et al. 2007), and numerous comparative genomic analyses including the small number of rosid genome sequences available in 2012 (for review, see Salse 2012), an ancestral hexaploid karyotype (21 = 3 × 7) deriving from a diploid progenitor with seven chromosomes was proposed based on the identification of seven triplets of homoeologous regions conserved between the genomes investigated. We used an entirely automated method for accurate reconstruction of the rosid ancestor in terms of ancestral gene order and content based on 1) the identification of orthologous and paralogous gene pairs on the basis of CIP/CALP alignment criteria, 2) the identification of synteny groups with Closeup software, 3) the reconstruction of CARs based on conserved gene adjacencies with ANGES software, and 4) the inference of preduplication CARs on the basis of DCS detection. In this method, threshold parameters are introduced only when defining orthologous and paralogous pairs/blocks. They are not required for ancestral genome reconstruction, which is based solely on the conservation of gene adjacencies within CARs. We used this new method to determine the chromosomal structure of the rosid ancestor (ARK) and of the ancestral Salicaceae, Rosaceae, Papilionoideae, Malvales, Brassicaceae, and Caricaceae intermediate karyotypes. By comparing modern rosid genomes, we were able to reconstruct a set of at least 6,250 protogenes for 21 and 7 protochromosomal groups, corresponding to the paleohexaploid ancestor (postpolyploidization karyotype) and its diploid (prepolyploidization karyotype) progenitor dating back to 150–250 Ma. The reconstructed ARK may largely underestimate the ancestral rosid gene repertoire, due to methodological limitations and the use of the grape genome as a reference in our study, but it nevertheless made it possible for us to investigate the paleohistory of modern rosid genomes in terms of chromosome and gene shuffling events. Finally, it has been shown that the shared ancestral γ WGD occurred in the common ancestor of rosids and asterids (Jiao et al. 2012). Our post-γ ARK may therefore be considered as the putative ancestral genome of eudicots in general, rather than specifically of rosids.

Modern rosids developed from the reconstructed ARK through a general phenomenon of chromosome number reduction, based on ancestral chromosome fusion and fission events. Most of the characterized ancestral chromosome fusions in rosids are telomeric chromosome fusions, contrasting with the centromeric chromosome fusions predominating in grasses (Murat et al. 2012). We can assume that the telomeric fusion of ancestral chromosomes in rosids gradually led to the evolution of dicentric chromosome intermediates, with one centromere becoming nonfunctional in modern monocentirc chromosomes. In addition to ancestral ARK chromosome fusions, rosids (grape, papaya, cacao, and strawberry with 1R; poplar, apple, and lotus with 2R; and finally *Arabidopsis* and soybean with 3R) have undergone lineage-specific polyploidization events during their history: ρ (7–15 Ma), α and β (53–69 Ma), and γ (115–138 Ma). The ancestral γ paleohexaploidization event is associated with the Jurassic/Cretaceous transition, during which species extension is known to have occurred, whereas the ρ, α, and β WGD events are associated with the more recent Paleogene and Neogene periods, during which the climate changed, becoming locally cooler and drier (Markgraf et al. 1995). The occurrence of WGDs at times of mass species extinction for largely unknown biotic reasons and during periods of climate change is consistent with the hypothesis that genome doubling acts as a source of innovation in biological functions, with the retained extra gene copies conferring phenotypic novelty (Fawcett et al. 2009). For example, the γ event that closely coincided with the rapid radiation of the core eudicot lineages may have favored the development of a more advantageous floral morphology through the documented duplication of the AP (Apetala) and SEP (Sepallata) gene families (Litt et al. 2003; Zahn et al. 2005).

Polyploidization has also been reported to be followed by the massive loss of duplicated genes, according to the subgenome dominance rule, as demonstrated principally in grasses (Schnable et al. 2012; Murat et al. 2014) and in a limited range of eudicots, including *Arabidopsis* (Ziolkowski et al. 2003), soybean (Henry et al. 2006), and *Brassica rapa* (Cheng et al. 2012). We established the ancestral nature of this phenomenon, by identifying orthologous dominant (i.e., higher levels of duplicated gene retention) and sensitive (i.e., higher levels of duplicated gene loss) chromosomal segments in modern rosids derived from the shared paleohexaploidization event, suggesting a shared prespeciation phenomenon. On the basis of the evolutionary fate of the 6,250 protogenes identified, in terms of their distribution between the 21 (post-γ ARK) and 7 (pre-γ ARK) protochromosomal groups, we developed a model of superimposed subgenome dominances between three progenitors—A (A3-4-8-10-14-16-20), B (A2-5-9-12-13-17-19), and C (A1-6-7-11-15-16-21)—clarifying the nature of the origin of the γ event. In this scenario, following a first hybridization event, subgenome A (dominant) retained most of the ancestral gene copies, whereas they were largely lost from subgenome B (sensitive); subgenome C was dominant over AB (tetraploid) in the framework of a second hybridization event, and this resulted in an allohexaploid ancestor with a genome structured into 21 chromosomes (Malacarne et al. 2012). The subgenome dominance phenomenon following the ancestral hexaploidization event in rosids is consistent with reports concerning the neohexaploidization of *Brassica rapa* (Tang et al. 2012) and *Triticum aestivum* (Pont et al. 2013), indicating that the hexaploid rosid ancestor (pre-γ ARK) was probably formed through two hybridization events. We suggest that, in all modern rosid genomes, the ancestral structural plasticity (or sensitivity) is partitioned into the genomic compartments inherited from protochromosomes A2-5-9-12-13-17-19. It would be interesting to investigate the role of such plastic compartments in driving responses to biotic and abiotic stresses in rosid crops. It has already been suggested that QTL (Quantitative Trait Locus) partitioning occurs after polyploidy, as only 21% of fiber quality QTLs in cotton (Rong et al. 2007) and 23% of fruit quality QTLs in strawberry (Lerceteau-Köhler et al. 2012) are located in homoeologous blocks. This suggests that the vast majority of QTLs are not maintained in the duplicated blocks, as a direct consequence of the diploidization mechanism. In the case of the recent polyploidization of *Brassica napus*, homoeologous loci may still be involved in resistance to stem canker (Fopa et al. 2014). Our results, and those of trait dissection studies, suggest a new hypothesis, requiring further investigation, according to which species adaptation traits (particularly those governing responses to biotic and abiotic stresses) may be partitioned between the currently defined dominant and sensitive chromosomal compartments inherited from ancient polyploidization events in crop genomes.

Paleogenomics data for rosids are available from a user-friendly online visualizer tool named DicotSyntenyViewer (available from http://urgi.versailles.inra.fr/synteny-dicot, last accessed February 13, 2015), which constitutes a platform for 1) validating gene models considered suspect due to annotation errors, on the basis of the presence of several orthologous genomic regions in multiple species; 2) identifying patterns of conservation and divergence within coding regions or even conserved noncoding sequences; and 3) transferring genomic information from one species to a less well-studied taxon. The DicotSyntenyViewer platform can be used 1) to identify conserved orthologs in rosids on the basis of a sequence of interest (starting with a gene name), 2) to obtain a list of paralogs in rosids (conserved and duplicated regions from a single ancestral locus available on the same screen display), and 3) to evaluate locus synteny (a zoom in/out option providing, on the same screen display, physical windows corresponding to multiples of 10 genes). The DicotSyntenyViewer is a translational biology tool that automatically delivers a catalog of conserved orthologous sequences for any region of interest to support cross-genome (or syntenic) map-based cloning strategies (i.e., case examples from grases: Quraishi et al. 2009; Quraishi, Murat, et al. 2011; Quraishi, Abrouk, et al. 2011; Dibari et al. 2012) for transfer from models, such as *Arabidopsis thaliana,* to rosid relatives.

## Conclusions

The paleogenomic inference of rosid history revealed that the ARK was structured into 7 prochromosomes, containing 6,250 ordered protogenes. This ARK constitutes a unique resource for fundamental (i.e., providing a novel two-step evolutionary theory leading to the establishment of dominant [stable] and sensitive [plastic] genomic compartments in modern rosid crops) and applied (i.e., providing the DicotSyntenyViewer tool for accurate translational genomics in rosids) research purposes.

## Supplementary Material

Supplementary tables S1–S5 are available at *Genome Biology and Evolution* online (http://www.gbe.oxfordjournals.org/).

Supplementary Data
