# Supplementary material for: Karyotype and Gene Order Evolution from Reconstructed Extinct Ancestors Highlight Contrasts in Genome Plasticity of Modern Rosid Crops
Source: Genome Biol Evol. 2015 Jan 28;7(3):735–49. doi: 10.1093/gbe/evv014 (PMC5322550; doi:10.1093/gbe/evv014)
Supplement: Supplementary Data [file supp_7_3_735__index.html]

Karyotype and Gene Order Evolution from Reconstructed Extinct Ancestors Highlight Contrasts in Genome Plasticity of Modern Rosid Crops — Supplementary Data 

# Karyotype and Gene Order Evolution from Reconstructed Extinct Ancestors Highlight Contrasts in Genome Plasticity of Modern Rosid Crops

## Supplementary Data

files

**Files in this Data Supplement:**

- Supplementary Data - xlsx file
- Supplementary Data - xlsx file
- Supplementary Data - xlsx file
- Supplementary Data - xlsx file
- Supplementary Data - xlsx file
- Supplementary Data - docx file
