# Supplementary material for: Karyotype and Gene Order Evolution from Reconstructed Extinct Ancestors Highlight Contrasts in Genome Plasticity of Modern Rosid Crops
Source: Genome Biol Evol. 2015 Jan 28;7(3):735–49. doi: 10.1093/gbe/evv014 (PMC5322550; doi:10.1093/gbe/evv014)
Supplement: Supplementary Data [file supp_evv014_New_Microsoft_Office_Word_Document.docx]

**Additional material**

**Supplementary Table S1**: Orthologous and paralogous blocks within ancestral chromosomes.

**Supplementary Table S2:** Orthologous and paralogous genes on the ancestral chromosomes.

**Supplementary Table S3:** Orthologous and paralogous genes in modern rosid species.

**Supplementary Table S4:** ARK gene content and related genes in modern rosid species.

**Supplementary Table S5:** Dating of rosid duplication and speciation events.
